# The new X-ray absorption fine-structure beamline with sub-second time resolution at the Taiwan Photon Source

**DOI:** 10.1107/S1600577521001740

**Published:** 2021-03-12

**Authors:** Chih-Wen Pao, Jeng-Lung Chen, Jyh-Fu Lee, Meng-Che Tsai, Chi-Yi Huang, Chao-Chih Chiu, Chao-Yu Chang, Liang-Chih Chiang, Yu-Shan Huang

**Affiliations:** a National Synchrotron Radiation Research Center, Hsin-Ann Road, Hsinchu Science Park, Hsinchu 30076, Taiwan; b NanoElectroChemistry Lab, Graduate Institute of Applied Science and Technology, National Taiwan University of Science and Technology, Taipei, Taiwan

**Keywords:** XAFS, QXAFS, *in situ*/*operando*

## Abstract

The new TPS 44A beamline at the Taiwan Photon Source, located at the National Synchrotron Radiation Research Center, is presented. This beamline is equipped with a new quick-scanning monochromator (Q-Mono), which can provide both conventional step-by-step scans (s-scans) and on-the-fly scans (q-scans) for X-ray absorption fine-structure (XAFS) spectroscopy experiments, including X-ray absorption near-edge structure (XANES) and extended X-ray absorption fine-structure (EXAFS) spectral measurements. Ti and Te *K*-edge XAFS spectra were used to demonstrate the capability of collecting spectra at the limits of the working energy range. The Ni and Cu *K*-edge XAFS spectra for a Cu-doped Pt/Ni nanocomposite were acquired to test the performance of the newly commissioned beamline. Pt *L*_3_- and Ru *K*-edge quick-scanning XAFS (QXAFS) spectra for standard Pt and Ru foils, respectively, revealed the stability of the q-scan technique. The results also demonstrated the beamline’s ability to collect XAFS spectra on a sub-second timescale. Furthermore, a Zn_(s)_|Zn^2+^_(aq)_|Cu_(s)_ system was tested to indicate that the states of the Zn electrode could be observed in real time for charging and discharging conditions using an *in situ*/*operando* setup combined with QXAFS measurements.

## Abstract 2

The new TPS 44A beamline at the Taiwan Photon Source is presented. The beamline is equipped with a new quick-scanning monochromator (Q-Mono), which can provide both conventional step-by-step scans and on-the-fly scans for XAFS spectroscopy experiments, including XANES and EXAFS spectral measurements.

## 1. Introduction   

X-ray absorption fine-structure (XAFS) spectroscopy, which includes X-ray absorption near-edge structure (XANES) and extended X-ray absorption fine structure (EXAFS), is an important experimental method used at synchrotron radiation facilities. Over the last 40 years, this method has been widely applied to scientific research and industry applications (Lee *et al.*, 1981 ▸). The XAFS spectral measurements require scanning of X-ray photon energies over a range of 1000 eV; these photon energies are selected using a monochromator. Conventional monochromators are driven by stepper motors, and the absorption coefficients of each energy value are measured at every step. A full XAFS spectrum can be obtained in a time ranging from a few minutes to ten minutes, depending on the experimental setup and the sample type. Recently, a quick-scanning monochromator (Q-Mono) has been successfully developed, which allowed a single spectrum to be acquired in the sub-second time scale, in contrast to the traditional acquisition time which can take tens of minutes (Müller *et al.*, 2015 ▸; Khalid *et al.*, 2010 ▸; Nonaka *et al.*, 2016 ▸). Thus, this evolution of XAFS spectroscopy into ‘quick-scanning XAFS (QXAFS)’ provides a powerful tool for time-resolved XAFS measurements. The local environment of the absorbing atoms in a sample can be tracked in real time using an *in situ* setup combined with the QXAFS technique. This can potentially facilitate experimental measurements under *in situ* or *operando* environments for monitoring various processes such as the charge and discharge cycles of batteries and chemical processes in catalysts (Eslava *et al.*, 2016 ▸; Marchionni *et al.*, 2020 ▸; Gaur *et al.*, 2019 ▸).

The theory and first experiment using QXAFS was proposed and performed by Ronald Frahm in 1988 (Frahm, 1988 ▸). In typical XAFS experiments the spectra of Co and Fe standard foils can be obtained in a few seconds. Several quick-scanning beamlines have been built around the world over the last ten years to facilitate QXAFS measurements. The capability of quickly scanning the energy of X-ray photons has been achieved with different results via different designs of Q-Mono. For example, a group at NSLS developed a cam system driven by a DC motor that was installed at the X18B beamline (Khalid *et al.*, 2010 ▸). The spectrum can be collected both in the low to high (up spectrum) and the high to low (down spectrum) energy ranges, with each scan spanning 100 ms. The Toyota beamline at SPring-8 features a Q-Mono driven by high-speed AC servo motors, and it is capable of data acquisition in the sub-second range (Nonaka *et al.*, 2016 ▸). Recently, a Q-Mono with a direct drive and goniometer has been installed in the SuperXAS (SLS) and P64 Advance XAFS (DESY) beamlines. This enables very stable measurements in both the conventional step-by-step scan (s-scan) mode and the on-the-fly scan (q-scan) mode (Müller *et al.*, 2016 ▸; Bornmann *et al.*, 2019 ▸).

Experimental techniques such as energy-dispersive X-ray absorption spectroscopy (EDXAS) and pump–probe X-ray absorption spectroscopy (XAS) improve time resolution from the sub-second scale to the nanosecond scale or lower (Pascarelli *et al.*, 2016 ▸; Lima *et al.*, 2011 ▸; Smolentsev *et al.*, 2013 ▸). However, the EDXAS setup does not allow for measurements of the fluorescence signal in samples, and it requires a very flat sample surface. Pump–probe XAS can only be used for samples that can be excited by pulse laser, such as photocatalysts (Uemura *et al.*, 2020 ▸; Smolentsev *et al.*, 2018 ▸). The QXAFS technique can be more flexible and convenient for *in situ*/*operando* XAFS measurements. We have developed and constructed a QXAFS beamline (TPS 44A) using a bending-magnet source at the newly constructed Taiwan Photon Source (TPS). This beamline has been commissioned to provide a highly flexible and stable experimental facility that permits the acquisition of QXAFS and conventional XAFS spectra.

## 2. Beamline   

**Figure 1:**
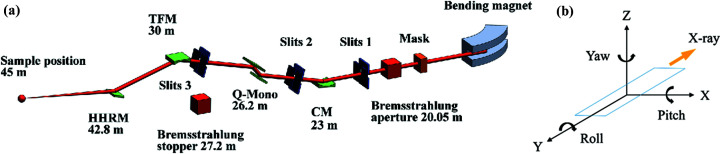
(*a*) Schematic of the optical design configuration, where CM is a collimating mirror, Q-Mono is a quick-scanning monochromator, and TFM is a toroidal focusing mirror. (*b*) The coordinate axes of the system.

The TPS 44A beamline is designed to provide an X-ray photon source with a stable and wide energy range for QXAFS measurements. The bending magnet is therefore used as a photon source for this beamline. The optical components include a bendable collimating mirror (CM), a channel-cut Q-Mono, two bendable toroidal focusing mirrors (TFMs), and a high-harmonic-rejection mirror (HHRM). To measure the *K*- or *L*-edge XAFS spectra of the elements in the periodic table to the maximum extent possible, we set the working energy of this beamline to range from 4.5 to 34 keV. The construction and commissioning of the beamline were completed at the end of 2018. The optical layout and the coordinate axes are displayed in Fig. 1 ▸. In addition, Table 1 ▸ shows the beamline specification.

### 2.1. Photon source   

The TPS machine is currently operating at the electron energy of 3 GeV (Horiuchi, 2015 ▸). The electron beam current is 400 mA in the top-up operation mode. The magnetic peak field and critical energy of the bending magnet is 1.198 T and 7.12 keV, respectively, and it is used as the photon source for the TPS 44A beamline. The low power density of the bending magnet is advantageous because it reduces the thermal deformation and improves the stability of the optics, with the broad spectrum requiring no complex control like undulator gap tuning. The feasible length of the TFM and its small sagittal curvature limit the photon acceptance of beamline, so the effective angular opening is 1 mrad × 0.11 mrad (H × V).

### 2.2. Collimating mirror   

The collimating mirror is the first optical device in this beamline. It is used to collimate the divergence of the bending-magnet radiation in the vertical direction so that the energy resolution can satisfy the experimental needs. To ensure radiation safety and the operational convenience of the beamline, some essential components are placed before the CM. Thus, the CM is placed 23 m from the source. The reflectivity of the mirror decreases with the grazing angle as well as energy. A smaller grazing angle results in higher reflectivity and a longer mirror length. Manufacturing high-quality long mirrors is a challenge. By setting the CM’s grazing angle to 2.5 mrad, the system can create a compromise between the feasible mirror length and the photon flux. The effective length of the CM is set to 100 cm, and the mirror faces upward. The CM is bendable, and the meridional radius is 18.4 km with a 2.5 mrad grazing angle. To minimize thermal deformations in the CM, a side water-cooling system is incorporated. To account for the high harmonic rejection, the mirror includes three reflection stripes, namely Si, Rh and Pt coating on a single silicon substrate.

### 2.3. Quick-scanning monochromator   

The Q-Mono is the key component of this beamline. It is located 26 m from the source, allowing it to select the photon energies via a channel-cut crystal with a Si (111) diffraction plane. The channel-cut crystal is driven by a torque motor mounted at the center of the goniometer, which enables the Q-Mono to perform conventional s-scans and q-scans for frequencies up to 50 Hz (Müller *et al.*, 2015 ▸). The s-scan XAFS measurements are performed using a Huber goniometer driven by the stepper motor with a step/encoder resolution of 5 × 10^−5^ degrees. The torque motor is driven by a sine wave signal and can scan smoothly in different angular ranges at different goniometer angles. The stage of channel-cut crystal is connected to the torque motor, to ensure rapid photon energy scanning. Although the angular scan range of the torque motor depends on the Bragg angle and the scanning speed, the maximum angular range of this motor can reach up to 4.2°, which can be used for energies scanning for 800 eV at Ti *K*-edge spectral measurements.

### 2.4. Toroid focusing mirrors   

**Figure 2:**
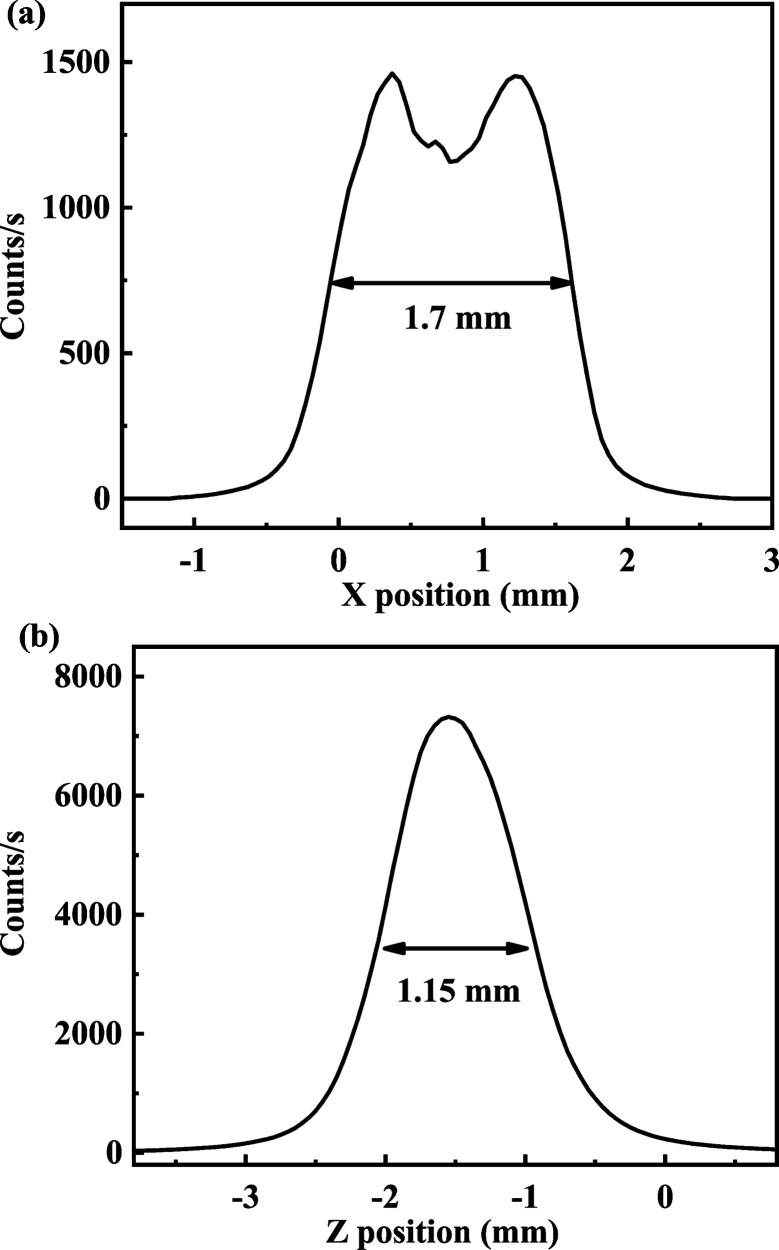
The X-ray beam profile of (*a*) the horizontal direction and (*b*) the vertical direction at the sample position. The sample position is 44 m from the photon source, and the focal point of the TFM is 45 m from the photon source. The curves were obtained at a photon energy of 8979 eV with slit openings of 10 µm and a scanning step of 10 µm.

**Figure 3:**
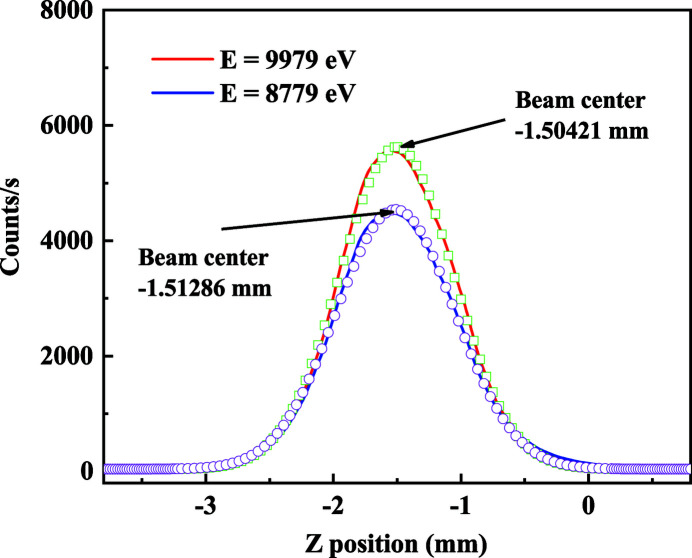
The vertical beam profile at the sample position. The blue and red lines are the profiles measured at a photon energy of 8779 eV and 9979 eV, respectively. The green squares and purple circles are the best Gaussian fitting.

This focusing mirror is placed 30 m from the source and is composed of two bendable toroid mirrors with Rh and Pt coatings, respectively. The grazing angle of the TFM is 2.5 mrad, like that of the CM. The focusing mirror has a demagnification ratio of 1:2 to minimize aberration in the horizontal direction. In the vertical direction, the CM and TFM form a focal spot 45 m from the source. Finally, the X-ray beam is focused to an area of 100 µm × 300 µm (H × V) at the focal point of the TFM. The Q-Mono uses the channel-cut crystal to select the photon energy, so the distance between the two diffraction planes is fixed. Therefore, the beam offset of the Q-Mono varies with photon energy during the XAFS measurements. The vertical position of the TFM is therefore tailored for the change of the beam height. For operation convenience, the TFM is set at a moderate vertical position during the energy scan. Table 2 ▸ demonstrates the relationship between the photon energy range, beam offset and the variation in the beam offset. The maximum difference in the beam offset is approximately 700 µm during a 1000 eV energy scan. The sample position is placed 44 m from the photon source, and the beam size is 1.7 mm × 1.15 mm (H × V). The beam profile is shown in Fig. 2 ▸. Fig. 3 ▸ displays the beam profile in the vertical direction at photon energies of 8779 and 9979 eV. These two values are the starting and ending photon energy of the Cu *K*-edge XAFS spectrum. Fig. 3 ▸ shows that the beam center almost coincides during spectral measurements for Cu *K*-edge XAFS. The Gaussian fitting result shows that the variation in the beam offset between the photon energy of the starting and ending points of the Cu *K*-edge XAFS spectrum is 10 µm. According to this measurement and an optical simulation, the beam position at the sample position is not altered much, and the photon loss is acceptable for this difference in beam offset variation.

### 2.5. High-order harmonic rejection mirror   

**Figure 4:**
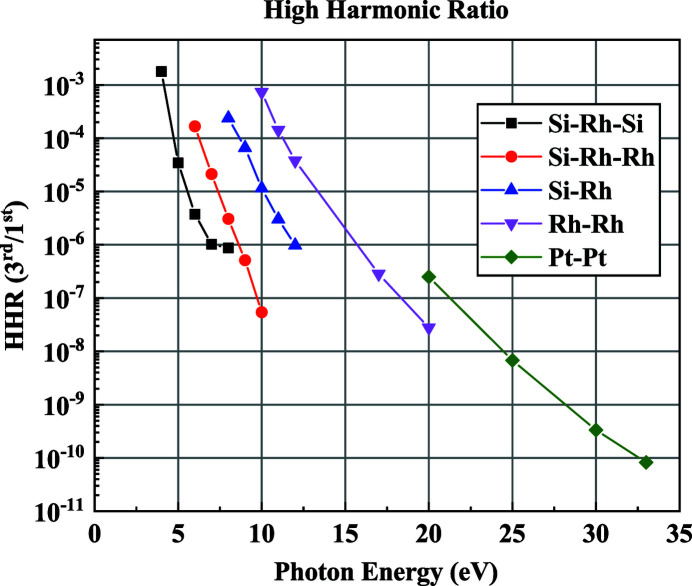
Harmonic rejection ratio (3rd/1st) at the sample position simulated by *SHADOW* with both the collimating mirror and the toroidal focusing mirrors set at a grazing angle of 2.5 mrad and with the HHRM set at 4.5 mrad.

The HHRM is used to reduce the contaminations of high-order photons at the working energy lower than 8 keV. Two important approaches for rejecting high-order harmonic photons include the crystal detuning of double-crystal monochromators and the use of grazing-incidence mirrors. Like for the beam offset variation issue, the crystal detuning method cannot be applied for the channel-cut crystal. In this setup, the grazing-incidence mirrors are used for high harmonics rejection (Hastings *et al.*, 1978 ▸; Latimer *et al.*, 1995 ▸; Lamble, 1995 ▸). For this beamline, the CM has three reflection surfaces, namely Si, Rh and Pt, and the coating surfaces of the two TFMs are Rh and Pt, respectively. To suppress the reflection of high harmonic photons, the corresponding reflective surface on the CM is selected in conjunction with the specific coating of the TFM. Under such an operation, a high harmonics rejection ratio (3rd/1st) lower than 10^−4^ can be achieved in the working energy above 8 keV. However, at low energies, it is still difficult to reach our goal using these combinations. An extra plane mirror with Rh and Si stripes was therefore inserted before the sample position to further suppress high harmonics. The plane mirror has a fixed grazing angle of 4.5 mrad and reflects upward. The harmonic ratios for different photon energies, simulated by the *SHADOW* (Sanchez del Rio *et al.*, 2011 ▸) software, are shown in Fig. 4 ▸. The harmonic content is reduced to 10^−4^ or less for photon energies greater than 4.8 keV. Based on the results of the simulation, five operation modes, shown in Table 3 ▸, are proposed, in which the mirror stripes are switched according to the working energy.

### 2.6. Beamline control   

The Experimental Physics and Industrial Control System (EPICS) was used as the control system for this beamline. Almost all beamline components, such as motors, counters and various equipment controllers, are controlled by EPICS, which provides low-level instrumentation of the beamline components by interfacing various electronic hardware components, including motor controllers, screen-monitoring CCDs, and current amplifiers. There are several high-level data collection Python scripts based on EPICS that can be used to describe experimental procedures.

## 3. Experimental end-station and beamline performance   

The experimental end-station is dedicated to measuring the XAFS spectrum in the s-scan and q-scan modes. All components are placed on a motorized optical table. A set of *X–Z* slits is located at the front of the optical table and is used to select the central portion of the mono X-ray beam. The slits can minimize the effect of the beam offset variations associated with photon energy on the data quality of the XAFS spectrum. The rise time of the gridded ionization chamber is roughly two orders of magnitude faster than comparable parallel plate ionization chambers (Müller *et al.*, 2013 ▸). The gridded ionization chambers are therefore used to detect the photon flux for both the q-scan and s-scan modes. ISEG THQ-series high voltage power is supplied with a two-output channel and is used to create a potential on the cathode and grid. The ionization chamber is always operated at saturation voltage. The output signal of the gridded ionization chamber is collected using a Keithley 428 current amplifier. This setup is used to perform the XAFS spectral measurements in the transmission mode. To obtain the QXAFS spectra in the fluorescence mode, a pin diode is used as the fluorescence detector. For samples with very low elemental concentrations (a few to several thousand p.p.m.), the spectra were obtained using a seven-element silicon drift detector (SDD) in the s-scan mode. For the s-scans, the voltage signals from the current amplifiers are converted into frequencies using voltage-to-frequency converters (Tsuji Electronics VF8-01) and digitized using a counter (Tsuji Electronics CT08-01E). The procedure for the s-scan data collection is described by Python scripts based on EPICS. The data acquisition system (DAQ) for the q-scan mode is separate from the beam control system (Stötzel *et al.*, 2011 ▸; Müller *et al.*, 2016 ▸). The acquired signals are digitized using a 16-bit analog-to-digital converter board (National Instruments PXIe-6366) with a maximum sampling rate of 2 MS s^−1^ per channel. The signals for the angular encoders on the Q-Mono are recorded simultaneously. The NIPXIe-1073 chassis includes a 10 MHz reference clock, which can perfectly synchronize all devices in the DAQ. Once the HHRM reduces the high-order photons contamination, the mono X-ray beam refracts upwards by 9 mrad. The height and pitch angle of the optical table can be optimized to maintain the relative position of the desktop and X-ray beam.

**Figure 5:**
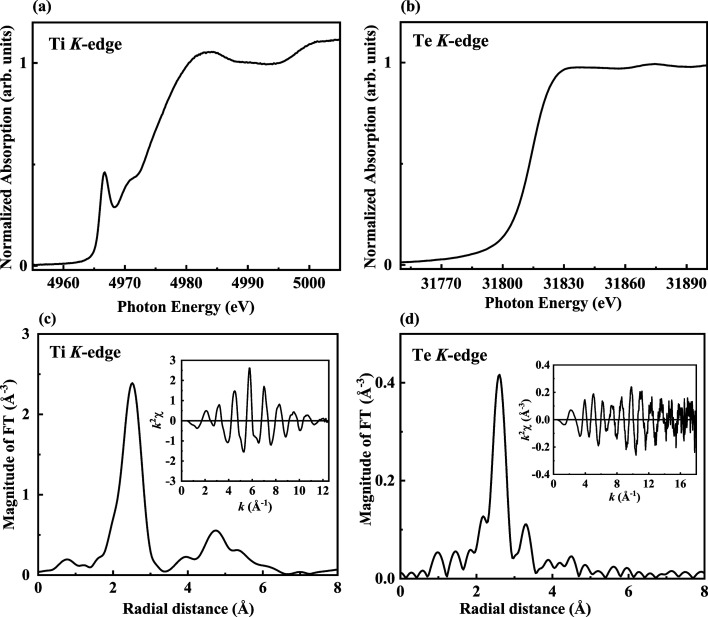
Normalized Ti (*a*) and Te (*b*) *K*-edge XANES spectra of Ti standard foil and Te powder. Panels (*c*) and (*d*) are the Fourier transform (FT) spectra of Ti standard foil and Te powder at the *K*-edge EXAFS spectra. The insets in (*c*) and (*d*) show the EXAFS *k* ^2^χ data. The FT range is 3–12 Å^−1^ and 4–15 Å^−1^ for the Ti and Te *K*-edge EXAFS spectra, respectively. The data were averaged through 120 spectra obtained in q-scan mode at a Q-Mono oscillation frequency of 1 Hz.

The working energy range of this beamline spans from 4.5 to 34 keV, which is a range suitable for researching topics related to titanium and tellurium elements. Fig. 5 ▸ presents the results of *K*-edge absorption measurements for Ti standard foil and Te powder. This figure demonstrates that the TPS 44A can collect spectra across the range from the lowest to highest photon energies. The spectra were obtained in transmission mode using q-scan at a Q-Mono oscillation frequency of 1 Hz, and 120 spectra were averaged together. The following two cases were used to test the performance of the q-scan mode.

### 3.1. Cu-doped Pt/Ni nanocomposite   

**Figure 6:**
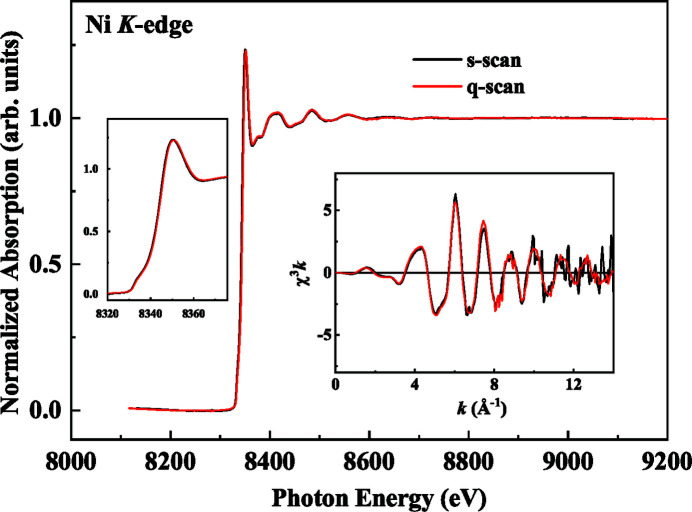
Normalized Ni *K*-edge XAFS spectrum of a Cu-doped Pt/Ni nanocomposite obtained using the traditional s-scan (black) and the q-scan (red) methods. The left inset shows a zoomed-in image of the XANES range, and the right inset presents the *k* ^3^-weighted EXAFS spectra. The q-scan data were obtained by averaging 120 spectra at a Q-Mono oscillation frequency of 1 Hz.

We acquired the Ni *K*-edge XAFS spectra of a Cu-doped Pt/Ni nanocomposite to test the performance of the q-scan experimental method. The sample contained Pt, Cu and Ni with elemental concentrations of 14, 0.37 and 2.71 wt%, respectively. Fig. 6 ▸ displays the normalized Ni *K*-edge XAFS spectra obtained using the s-scan and q-scan methods in the transmission mode. The q-scan data were collected using a Q-Mono oscillating at 1 Hz for 1 min. Two complete spectra were acquired per second, resulting in 120 averaged spectra with an improved signal-to-noise (S/N) ratio, as illustrated in Fig. 6 ▸. By contrast, the time to obtain one spectrum in the s-scan mode was approximately 30 min compared with 1 min required by the q-scan mode. The resultant spectra of the two modes perfectly overlapped in both the XANES and EXAFS regions, indicating the reliability and reproducibility of the q-scan method. Therefore, we successfully developed a q-scan technique that resulted in a significant decrease in the data collection time, improving the operating efficiency of the beamline. The rapid data acquisition rate can also reduce radiation-induced damage in biological research; thus, this technology facilitates investigations in new areas of research. The right-hand inset of Fig. 6 ▸ shows the Ni *K*-edge *k* ^3^-weighted EXAFS spectrum of a Cu-doped Pt/Ni nanocomposite. The spectra collected using the q-scan method are considerably smoother than those collected using s-scan, particularly in the region in which the *k*-value is greater than 8, indicating that the q-scan experimental method can generate better quality data in addition to increasing the rapidity of the data acquisition.

**Figure 7:**
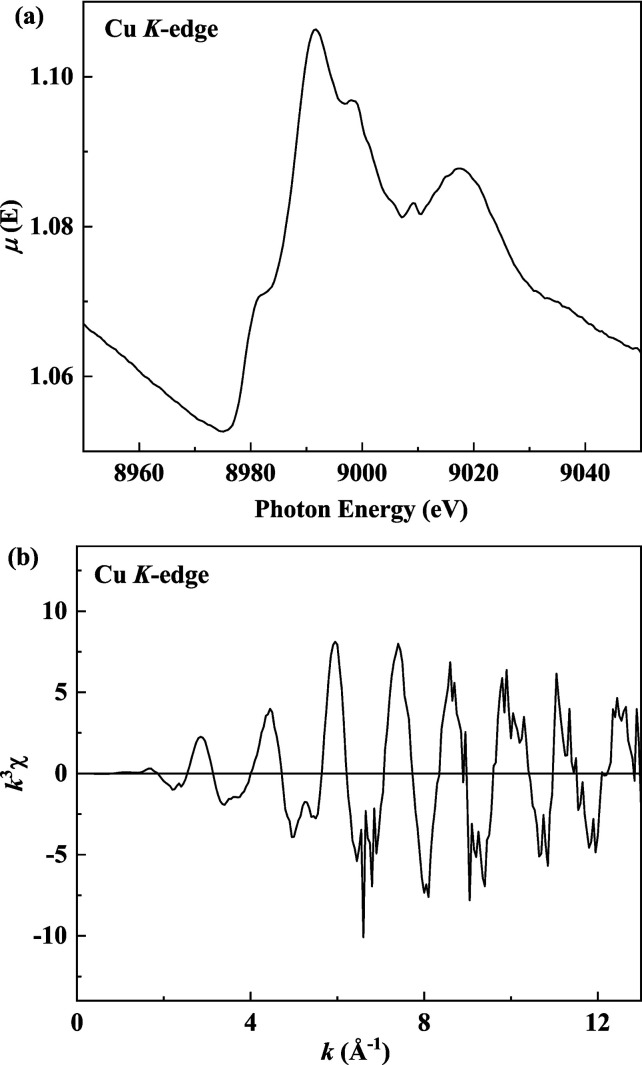
The Cu *K*-edge (*a*) XANES and (*b*) *k* ^3^-weighted EXAFS spectra of the Cu-doped Pt/Ni nanocomposites.

Conventional methods such as transmission and fluorescence XAFS are used to measure samples having high and low elemental concentrations, respectively (Bunker, 2010 ▸). In general, the transmission mode is not suitable for investigating a sample with a white line edge-jump smaller than 0.1. Fig. 7 ▸(*a*) presents the Cu *K*-edge XANES spectrum of the Cu-doped Pt/Ni nanocomposite after 1 min in the transmission mode using the q-scan acquisition method. Although the white line edge-jump of the absorption coefficient was only 0.03, the XANES region showed a highly smooth spectrum. The EXAFS oscillations exhibit a clear phase and strong amplitude, as illustrated in Fig. 7 ▸(*b*), indicating that the data are suitable for EXAFS processing. XAFS measurements in samples with elemental concentrations less than 1 wt% should be performed in the fluorescence mode using an SDD or a Lytle detector. However, because of the high intensity of the Ni *K*_α_-emission line in this nanocomposite, the SDD detector will become saturated. By contrast, because there is background from the Ni *K*_α_-emission line, the S/N ratio of the spectrum collected using the Lytle detector is expected to be very poor. Therefore, the transmission mode using the q-scan method can be used to directly obtain the data, resulting in more rapid and effective measurements. This study demonstrates that the q-scan experimental method can improve the detection limit of XAS transmission measurements.

### 3.2. Time-resolved XAFS   

**Figure 8:**
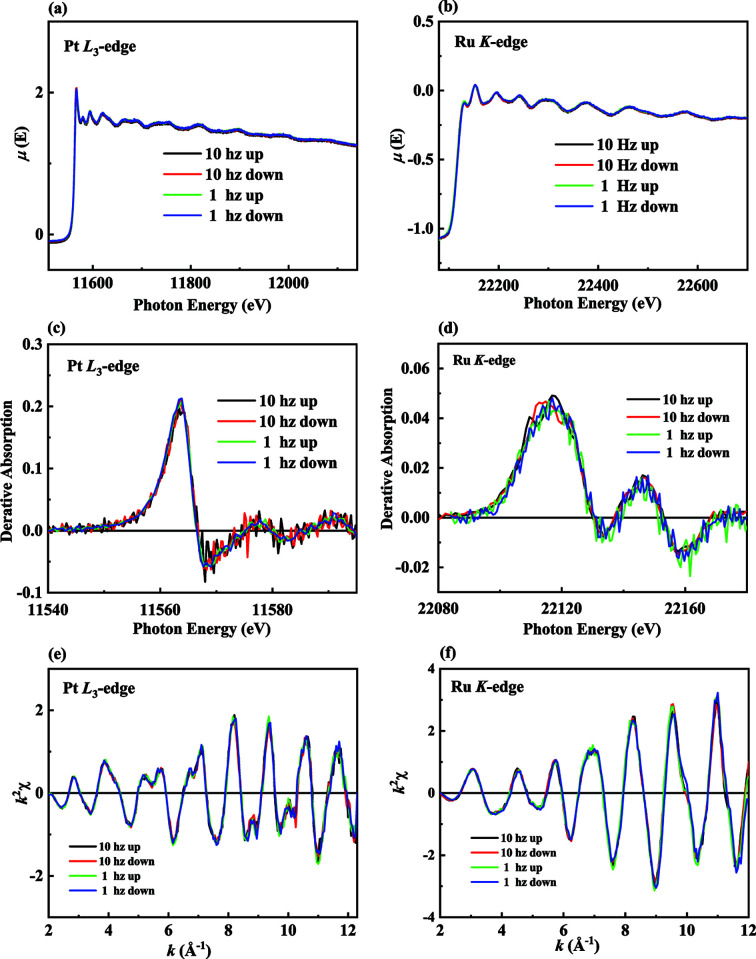
Single XAFS spectra of (*a*) Pt and (*b*) Ru standard foil at the Pt *L*_3_-edge and Ru *K*-edge at Q-Mono oscillation frequencies of 1 and 10 Hz, respectively. The up spectrum (black line) represents the sweep of the photon energy from low to high values, while the down spectrum (red line) shows the reverse trend. The normalized derivative of the XANES spectra for the (*c*) Pt *L*_3_-edge and (*d*) Ru *K*-edge. The *k* ^2^-weighted EXAFS data for the (*e*) Pt *L*_3_-edge and (*f*) Ru *K*-edge.

The QXAFS experimental method was developed mainly to provide a time-resolved technique for studying chemical processes on the sub-second timescale. Studies of chemical reactions, such as the oxidation/reduction process and ion exchange/absorption, are important to researchers in the pure and applied sciences. As shown in Fig. 8 ▸, the Pt *L*_3_-edge and Ru *K*-edge XAFS spectra of standard Pt and Ru foils, respectively, were acquired to demonstrate the stability of the proposed q-scan experimental method. The up/down spectra collected at frequencies of 1 and 10 Hz exhibit excellent reproducibility in both the XANES and EXAFS regions, demonstrating that the TPS 44A beamline now allows users to obtain data with a sub-second time resolution. In the XANES regime, a single spectrum can be collected in 10 ms due to the shorter scanning range (∼200 eV) compared with the EXAFS regime.

**Figure 9:**
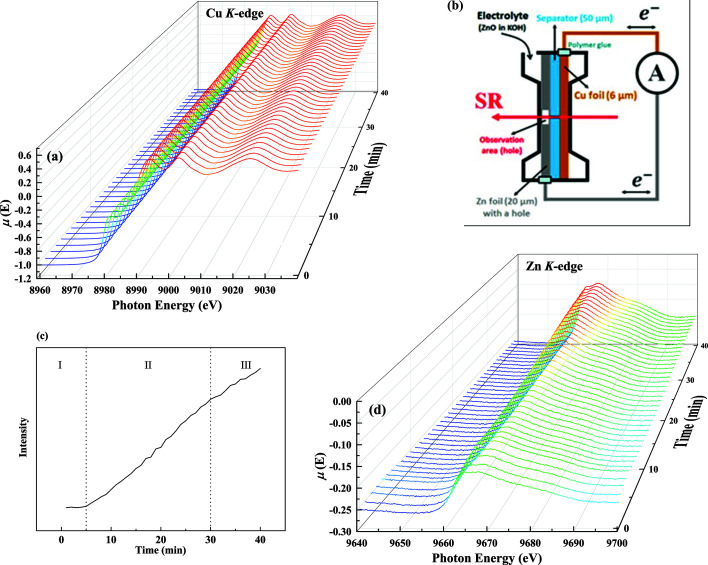
The *in situ* measurements were performed at a Q-Mono oscillation frequency of 1 Hz. (*a*) The Cu *K*-edge time-dependent XANES spectra of the Zn_(s)_|Zn^2+^_(aq)_|Cu_(s)_ system. (*b*) Schematic of the *in situ* measurement setup. (*c*) Time dependence of the Zn *K*-edge XANES region after subtracting the background from the Cu atom signal. (*d*) The Zn *K*-edge time-dependent XANES spectra of the Zn_(s)_|Zn^2+^_(aq)_|Cu_(s)_ system.

An *in situ* test measurement of a Zn_(s)_|Zn^2+^_(aq)_|Cu_(s)_ system during Zn plating/stripping on Cu was performed at a 1 Hz oscillation frequency, with the experimental setup and results shown in Fig. 9 ▸ (Wang *et al.*, 2018 ▸; Shin *et al.*, 2016 ▸). The Cu *K*-edge and Zn *K*-edge XAS spectra were measured using a single scan with a photon energy range of 2000 eV by optimizing the oscillation range of the Q-Mono. While clear features corresponding to Cu metal can be observed in the Cu *K*-edge XANES spectra [Fig. 9 ▸(*a*)], the Zn *K*-edge XANES spectra [Fig. 9 ▸(*d*)] show an increase in the unoccupied 4*p* states of the Zn atoms during Zn plating/stripping. Figure 9 ▸(*c*) shows the peak intensity at a photon energy of 9667.5 eV in Zn *K*-edge XANES spectra after standard data processing through the *Demeter* software package (Ravel & Newville, 2005 ▸). The monotonically increasing intensity contains three different slopes (regions I, II and III), indicating the existence of three different Zn atom states at the Cu electrode. The plating and stripping of Zn on the Cu foil were carried out at a constant density of 1 mA cm^−2^ (plating step duration: 25 min; stripping step duration: 15 min). The first region corresponds to 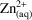 in the electrolyte, the second region includes both 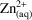 and deposited Zn in the plating step, while the third corresponds to the dissolution of the deposited Zn during the stripping step. We observed a Coulombic efficiency less than 100%, indicating that Zn was not completely stripped from the Cu surface. Therefore, the spectra were not recovered to their original state. This test case showed that the states of the Zn electrode could be observed in real time under charging and discharging conditions via an *in situ*/*in operando* setup combined with QXAFS measurements. The experimental results provided critical information about the reaction dynamics within battery devices, which cannot be obtained through conventional XAFS measurement methods.

## 4. Summary   

The TPS 44A beamline now has the ability to facilitate conventional s-scan and q-scan experimental methods for XAFS studies. The q-scan technique not only enables fast data acquisition but also improves the data quality. The combination of an *in situ*/*in operando* setup and the QXAFS measurements provides a new powerful tool for time-resolved studies, which is useful for energy materials research and for observing chemical reactions on the sub-second timescale. This QXAFS technique can provide new scientific research opportunities to XAS users. Additionally, the beamline can serve as an important facility for both basic science and industrial/applied research studies.

## Figures and Tables

**Table 1:** Beamline specifications of TPS 44A

Energy range	4.5–34 keV
Beam spot at focal point	100 µm (H) × 300 µm (V)
Beam spot at sample position	1.7 mm (H) × 1.15 mm (V)
Photon flux	3 × 10^11^ photons s^−1^ at 10 keV†
Q-Mono	Channel-cut Si(111) crystal
Energy resolution	1.4 06 × 10^−4^ at 5 keV‡
High harmonic rejection ratio	<10^−4^
Time to collect single spectrum	50/10 ms (EXAFS/XANES)

† Measured with 500 mA top-up mode in the storage ring.

‡ The value is obtained from the result of *SHADOW* calculations.

**Table 2:** Relationship between photon energy range, beam offset and the variation in the beam offset in the vertical direction

Energy range (keV)	Beam offset (mm)	Beam offset variation (µm)
4.5–5.5	17.068–17.730	649
5.5–7.5	17.730–18.328	598
7–11	18.226–18.691	465
10–34	18.625–18.968	343

**Table 3:** Selection of reflective stripes according to photon energies

Operation energy range (keV)	CM (2.5 mrad)	TFM (2.5 mrad)	HHRM (4.5 mrad)
4.8–7	Si	Rh	Si
6.2–12	Si	Rh	Rh
8.2–12	Si	Rh	
10.5–23	Rh	Rh	
22–34	Pt	Pt	
